# Impact of COVID-19 Pandemic on Hospital Admissions of Acute Coronary Syndrome: A Beijing Inpatient Database Study

**DOI:** 10.1016/j.lanwpc.2021.100335

**Published:** 2021-12-11

**Authors:** Liu He, Feng Lu, Xin Du, Deyong Long, Caihua Sang, Ribo Tang, Jianzeng Dong, Moning Guo, Changsheng Ma

**Affiliations:** a Department of Cardiology, Beijing Anzhen Hospital, Capital Medical University, National Clinical Research Center for Cardiovascular Diseases, Beijing Advanced Innovation Center for Big Data-Based Precision Medicine for Cardiovascular Diseases, Beijing, China; b Beijing Municipal Health Commission Information Center, Beijing, China; c Heart Health Research Center, Beijing, China; d The George Institute for Global Health, The University of New South Wales, Sydney, Australia

**Keywords:** Acute coronary syndrome, Heart failure, CoOVID-19 pandemic

## Abstract

**Background:**

Consequences of reduced acute coronary syndrome (ACS) admissions during COVID-19 pandemic periods were reported by different countries. However, admissions, treatments, and prognosis of ACS during and after COVID-19 pandemic in Beijing, China was unknown.

**Methods:**

Information on ACS admissions and heart failure (HF) admission were identified from database of Beijing Municipal Health Commission Information Center. Study period was defined as December 1, 2019 to June 30, 2020, and control period was defined as December 1, 2018 to June 30, 2019. Numbers of admission for HF during the control period, the study period, and seven months after study period were compared to evaluate the consequence of changed ACS care during the COVID-19 pandemic.

**Findings:**

Admissions for ST-elevation myocardial infarction (STEMI), Non-ST-elevation myocardial infarction (Non-STEMI), and unstable angina (UAP) reduced by 38·0%, 41·0%, and 63·3% (*N* = 1953, 1991, 7664 between January 24, 2020 to June 30, 2020 vs. *N* = 3150, 3373, and 20,868 between January 24, 2019 to June 30, 2019) in study period. Percutaneous coronary intervention performed within 24 h were significantly more frequent during study period in patients with STEMI (37·9% vs. 31·7%, *P*<0·0001), but significantly less frequent in patients with Non-STEMI (7·9% vs. 9·5%, *P* = 0·049), and in patients with UAP (1·7% vs. 3·5%, *P*<0·0001). In-hospital mortality rates in patients with ACS were similar during the study period and the control period (3·1% vs 2·5%, *P* = 0·174 for STEMI; 2·7% vs 2·3%, *P* = 0·429 for Non-STEMI; 0·2% vs 0·1%, *P* = 0·222 for UAP). A fall by 23.9% for HF admissions was also observed during the seven months following the study period than equivalent period in 2019.

**Interpretation:**

During COVID-19 pandemic, ACS admissions reduced significantly in Beijing; however, increase of HF admissions was not observed within seven months post-pandemic period, implying the pandemic didn't deteriorate the short-term prognosis for ACS.

**Funding:**

the National Natural Science Foundation of China (82,103,904), the National Key Research and Development Program of China (Grant number: 2020YFC2004803).

**Research in Context**

**Evidence before this study**

Dramatic reduction in patients admitted with acute coronary syndrome (ACS) were observed globally in the outbreak of COVID-19. Delays in treatments and reduced proportion of patients undergoing primary percutaneous coronary intervention (PCI) among ST segment elevated myocardial infarction (STEMI) patients were reported from several countries (e.g., the USA, England, and China) during the early phase of the pandemic. Although recent data from China's Disease Surveillance Points system showed non-significantly decrease in observed mortality rates of myocardial infarction in 2020 than predicted, it was concerned that patients who survive these delayed presentations of ACS will present later with more severe diseases, such as heart failure (HF).

**Added value of this study**

Our study showed, in spite the outbreak was quickly under control by strict measures in Beijing, profound declines in hospitalizations of STEMI, Non-ST-elevation myocardial infarction (Non-STEMI) and unstable angina (UAP) was observed by 38·0%, 41·0% and 63·3% from January 24, 2020 to June 30, 2020, in comparison with the same time period in 2019. Although numbers of cardiac procedures dropped drastically, proportion of 24-hour PCI was higher among STEMI patients in COVID-19 pandemic period than previous year. In-hospital mortality rates for either STEMI, Non-STEMI or UAP patients between pandemic and control periods were not significantly different, and subsequent increase of HF admissions was not observed, and on the contrary, it was 23.9% lower in July 1, 2020 to January 23, 2021 than equivalent time the year before. The current study indicated the COVID-19 pandemic didn't deteriorate the short-term prognosis of ACS patients in Beijing.

**Implication of all the available evidence**

The COVID-19 pandemic had impact on accessibility to in-hospital treatments for ACS patients, however, during the global outbreak of COVID-19, it didn't cause excess burden of cardiovascular disease in Beijing, China. Promote, active and strict strategies which were taken by the government and were strictly followed by the public, as well as high performance of patient care which was maintained by medical staff, might all make significant contributions to ensure patients get efficient health care during public health emergencies.

Alt-text: Unlabelled box

## Introduction

Dramatic fall of acute coronary syndrome (ACS) admissions was observed in many countries since the outbreak of COVID-19,^1^, ^2^, ^3^ as a result of reluctance of patients in seeking medical help or difficulties in accessing healthcare. Admissions to intensive cardiac care units (ICCUs) for ST segment elevated myocardial infarction (STEMI) and Non-ST-Elevation Myocardial Infarction (Non-STEMI) decreased by 33% and 61% in Italian during the first month after national lockdown, compared with the month before.^4^ A substantial decline of STEMI admissions, delays in treatment, and reduced proportion of patients undergoing primary percutaneous coronary intervention (PCI) in Chinese hospitals between December 27, 2019 and February 20, 2020 were reported.^5^ The postponement of procedures in Acute Myocardial Infarction (AMI) is concerned and it is worried that patients who survived these delayed treatments will present later with more severe diseases, such as heart failure (HF).^6^ A recent study showed mortality rates of myocardial infarction and hypertensive heart disease did not increase during 2020 in China,^7^ it's unknown whether the admissions of HF, as a common consequence of ACS, had been worsened since the pandemic.

In this study, we investigated the time trends of admissions for ACS and rates of cardiac procedures performed in patients with ACS before and during the COVID-19 pandemic in Beijing, China. We also investigated the time trend of admissions for HF before, during, and post pandemic periods.

## Methods

### Beijing inpatient database

The Beijing inpatient database was established by Beijing Municipal Health Commission Information Center since 2015, based on hospital information system. Data of hospitalization records from 31 tertiary hospitals in Beijing were uploaded into the Beijing inpatient database. Among all these hospitals, 24 hospitals could provide inpatient care to patients with ACS and HF, 18 hospitals have a facility of doing PCI, and 15 hospitals have a facility of doing coronary artery bypass grafting (CABG) procedures. Information on patient's characteristics, such as age, sex, insurance coverage, dates of admission and discharge or decease, primary diagnosis and comorbidities, interventions performed, as well as complications of intervention were automatically abstracted from electronic health care records. Diagnosis at discharge was coded according to the International Classification of Diseases 10th revision (ICD-10) codes.

### Data extraction

Data of ACS admissions, procedures received, and in-hospital death at the time period between December 1, 2019 to June 30, 2020 (study period) and the corresponding time period one year before (December 1, 2018 to June 30, 2019, control period) were identified from the Beijing admission database. The study period (212 days in total) included 53 observational days before the cordons sanitaire of Wuhan city (the date of January 23, 2020), when COVID-19 was first detected, and 24 days after the Beijing government reduced the public health emergency response to level Ⅲ (the date of June 6, 2020).

Diagnosis of ACS was classified into STEMI (ICD-10 codes: I21·0, I21·1, I21·2, I21·3), Non-STEMI (ICD-10 codes: I21·4), and unstable angina (UAP) (ICD-10 codes: I20·0, I20·1, I20·9). Cardiac procedures were extracted by identifying the International Classification of Diseases 9th revision Clinical Modification (ICD-9-CM) procedure codes. Codes of PCI (ICD-9-CM procedure codes: 00·66, 36·01, 36·02, 36·05, 36·06, 36·07, 36·09) and CABG (ICD-9-CM procedure codes: 36·10–36·19) were abstracted. PCI on the same day of admission was used as a surrogate of primary PCI as the exact time of reperfusion is not recorded in the system.

Ghali Charlson Comorbidity Index (CCI) score was calculated to assess the total burden of illnesses.^8^ Five comorbidities associated with mortality were included in the score: myocardial infarct (score=1, ICD-10 codes: I21·0, I21·1, I21·2, I21·3, I21·4), congestive heart failure (score=4, ICD-10 codes: I110, I130, I132, I50), peripheral vascular disease (score=2, ICD-10 codes: I70, K5), cerebrovascular disease (score=1, ICD-10 codes: G45, G46, I60–69), and moderate or severe renal disease (score=3, ICD-10 codes: N18·3-N18·6, Z9).

HF admissions were defined as ICD-10 codes: I50 in either primary or secondary diagnoses at discharge. Data of HF admissions from December 1, 2018 to January 23, 2021 were extracted. This time window included a 7-month lag than that used for ACS admissions, to see whether there were any changes in HF admissions after the dramatic drops in ACS admissions.

Our research protocol was approved by the Medical Ethics Committee of Beijing Anzhen Hospital, Capital Medical University. Informed consent was waived in this study.

### Statistical analysis

Day-to-day numbers of ACS admission, stratified by discharge diagnosis of STEMI, Non-STEMI, and UAP, were plotted along the timeline of major events related to COVID-19 in Beijing [The government activated the highest level of public health emergency response (Level Ⅰ) on Jan 24, 2020, reduced the level of public health emergency response (From Level Ⅰ to Level Ⅱ) on Apr 30, 2020, and further reduced the level of public health emergency response (From Level Ⅱ to Level Ⅲ) on Jun 6, 2020.]. Number and proportions of admitted STEMI, Non-STEMI, and UAP cases were presented by subgroups [sex, age groups (0–49, 50–59, 60–69, or ≥70 yrs.), and CCI score groups (0–2 or ≥3)]. Numbers and rates of procedures conducted, as well as rates of in-hospital mortality among patients admitted with STEMI, Non-STEMI, and UAP, were reported. Poisson models were used to estimate the 95% confidence interval (95% CI) of in-hospital mortality and to compare the difference between groups. The means (Standard Error, SE) of hospital length of stay during study period and control period were plotted by weeks and compared using *t-*test. Numbers of HF admission were plotted by days. Differences in numbers of admission or treatment rates before and after the pandemic were tested using χ^2^ test or Fisher's exact test. A two-sided *P* value less than 0·05 was considered statistically significant. All statistical analyses were performed using SAS software version 9·4 (SAS Institution Inc., Cary, NC, USA).

## Results

**Figure. 1:**
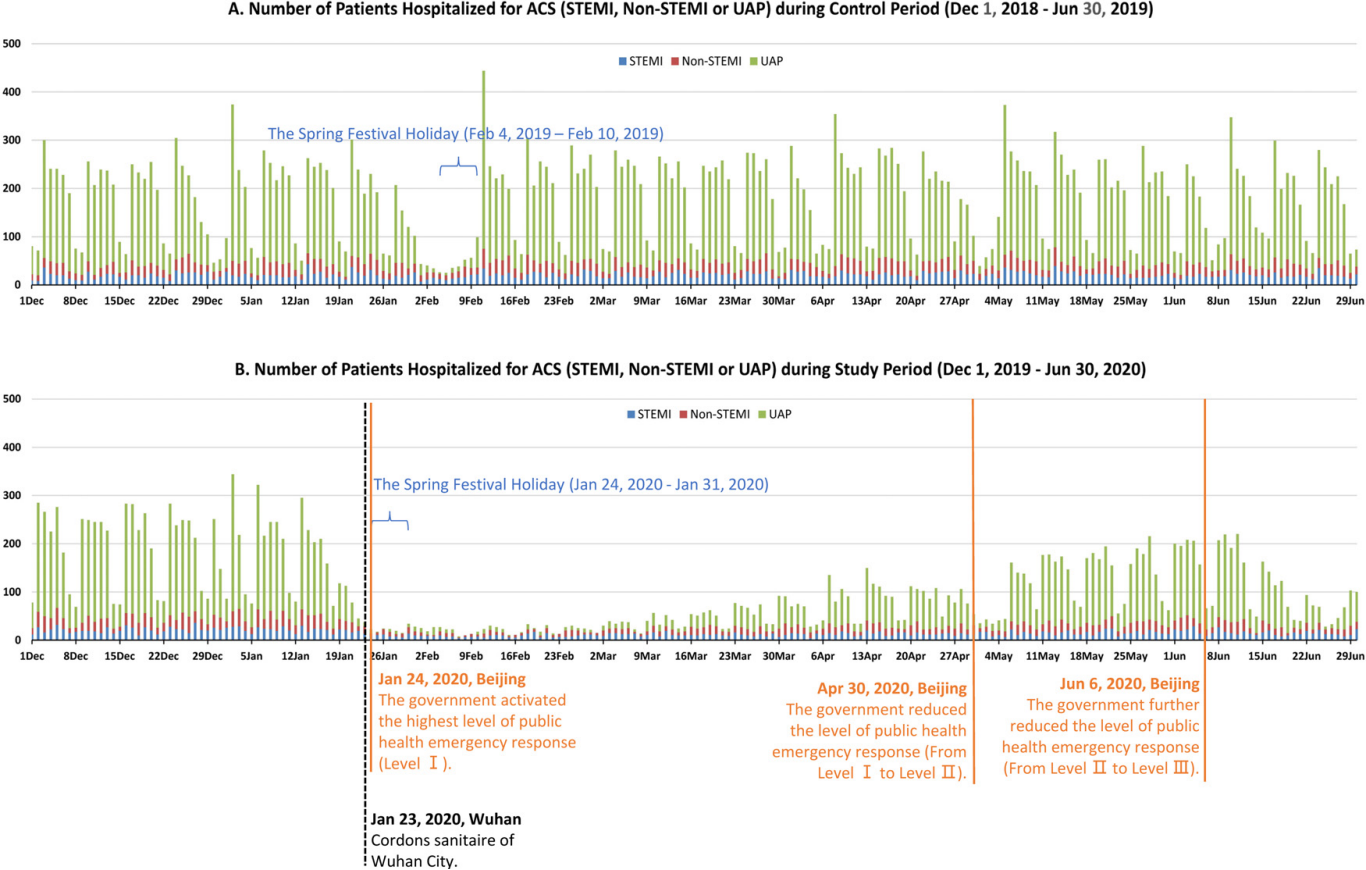
Trends of Admissions for ACS Patients during Study Period and Control Period. Numbers of patients admitted with STEMI, Non-STEMI, and UAP in Beijing reduced sharply since the government activated the highest level of public health emergency response (Level Ⅰ) on Jan 24, 2020, compared to the same period last year. Till June 30, 2020, ACS admissions remained low compared to the same time period in previous year, even after the Beijing government reduced the level of public health emergency response from the strictest Level I to Level II on May 11 2020,. **Abbreviations:** ACS, Acute Coronary Syndrome; STEMI, ST-Elevation Myocardial Infarction; Non-STEMI, Non-ST-Elevation Myocardial Infarction; UAP, Unstable Angina Pectoris; WHO, World Health Organization.

Numbers of patients admitted with STEMI (*N* = 1953), Non-STEMI (*N* = 1991), and UAP (*N* = 7664) between January 24, 2020 to June 30, 2020 reduced by 38·0%, 41.0%, and 63·3%, respectively, compared to the numbers of patients admitted with STEMI, Non-STEMI, and UAP during the same period last year (*N* = 3150, 3373, and 20,868 respectively). Till June 30, 2020, ACS admissions remained low compared to the same time period in previous year, even after the Beijing government reduced the level of public health emergency response from the strictest Level I to Level II on May 11 2020, (Fig. 1). In patients admitted with STEMI, Non-STEMI, and UAP during the study period, higher proportions of patients had a CCI scores≥3 (all *P*<0·0001 compared with patients admitted in previous year), and higher proportions of patients aged 60 years and above (*P*<0·05 in patients admitted with STEMI and in patients admitted with Non-STEMI), while proportions of females were not significantly different in patients admitted during the study period and the control period (Table 1).

**Table 1:** Numbers of Admissions (Proportions%) by Sex, Age Groups and CCI Scores for STEMI, Non-STEMI and UAP Patients during Study Period and Control Period*.

	STEMI			Non-STEMI			UAP		
	Study period	Control period	*P* value	Study period	Control period	*P* value	Study period	Control period	*P* value
Total No. of admissions	1953	3150		1991	3373		7664	20,868	
Female	429 (22·0%)	638 (20·3%)	0·144	532 (26·7%)	905 (26·8%)	0·930	2330 (30·4%)	6591 (31·6%)	0·056
Age groups									
0–49 yrs.	349 (17·9%)	591 (18·8%)	0·026	212 (10·6%)	443 (13·1%)	0·033	654 (8·5%)	1898 (9·1%)	0·390
50–59 yrs.	447 (22·9%)	816 (25·9%)		427 (21·4%)	747 (22·1%)		1891 (24·7%)	5223 (25·0%)	
60–69 yrs.	619 (31·7%)	966 (30·7%)		682 (34·3%)	1084 (32·1%)		3056 (39·9%)	8186 (39·2%)	
≥ 70 yrs.	538 (27·5%)	777 (24·7%)		670 (33·6%)	1099 (32·5%)		2063 (26·9%)	5561 (26·7%)	
CCI score ≥ 3	1130 (57·9%)	1542 (49·0%)	<0·0001	1126 (56·6%)	1711 (50·7%)	<0·0001	4085 (53·3%)	10,618 (50·9%)	<0·0001

⁎ Data before January 24, 2019 in control period and before January 24, 2020 in study period was excluded. Abbreviations: STEMI, ST-Elevation Myocardial Infarction; Non-STEMI, Non-ST-Elevation Myocardial Infarction; UAP, Unstable Angina Pectoris; CCI, Charlson Comorbidity Index.

**Figure. 2:**
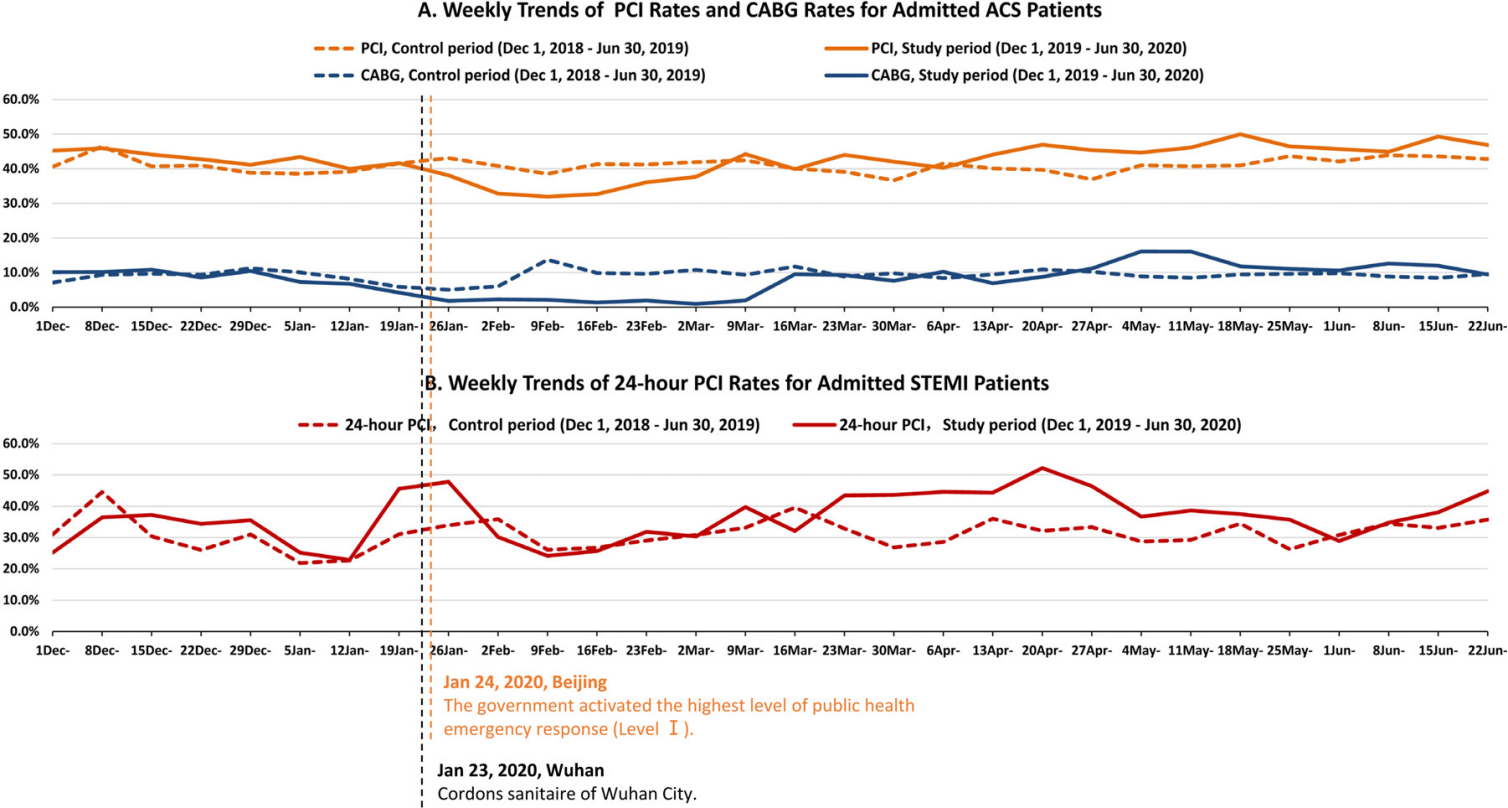
**Trends of Weekly Rates of cardiac procedures received in Patients Admitted with ACS during Study Period and Control Period.** Compared to the control period, weekly rates of PCI or CABG received in patients admitted with ACS reduced during the first month since the government activated the highest level of public health emergency response (Level Ⅰ) on Jan 24, 2020, but became higher since middle of March; the weekly rates of 24-hour PCI remained similar during the first two month since Jan 24, and much higher during the subsequent period. **Abbreviations:** ACS, Acute Coronary Syndrome; STEMI, ST-Elevation Myocardial Infarction; PCI, Percutaneous Coronary Intervention; CABG, Coronary Artery Bypass Grafting.

Proportions of ACS patients received PCI therapy were higher in the study period compared to that in the control period (56·7% vs. 51·6% in patients with STEMI, *P*<0·0001; 44·7% vs. 41·2% in patients with Non-STEMI, *P* = 0·012; 41·6% vs. 39·6% in patients with UAP, *P* = 0·002; Fig. 2). Proportions of PCI performed within 24-hour of admission were higher among STEMI patients in the study period (37·9% vs. 31·7%, *P*<0·0001), but lower among Non-STEMI patients (7·9% vs. 9·5%, *P* = 0·049) and UAP patients (1·7% vs. 3·5%, *P*<0·0001). A higher proportion of patients with UAP received CABG therapy (14·3% vs. 11·4%, *P*<0·0001) (Appendix p 1–2).

In-hospital mortality rates in patients with STEMI, Non-STEMI, and UAP admitted in the study period were similar with those admitted in the control period (Table 2), except a higher in-hospital mortality rate in male patients with STEMI (2·7%, 95% CI: 2·0%−3·7% vs. 1·7%, 95% CI: 2·0%−3·7%, *P* = 0·030; Table 2).

**Table 2:** Numbers [Rates%, 95% CI] of In-hospital Death for Admitted STEMI, Non-STEMI and UAP Patients during Study Period and Control Period.*.

	STEMI			Non-STEMI			UAP		
	Study period	Control period	*P* value	Study period	Control period	*P* value	Study period	Control period	*P* value
All	61 [3·1%, 2·4%−4·0%]	78 [2·5%, 2·0%−3·1%]	0·174	53 [2·7%, 2·0%−3·5%]	78 [2·3%, 1·9%−2·9%]	0·429	12 [0·2%, 0·1%−0·3%]	21 [0·1%, 0·1%−0·2%]	0·222
Sex									
Female	20 [4·7%, 3·0%−7·2%] #	36 [5·6%, 4·1%−7·8%] #	0·494	17 [3·2%, 2·0%−5·1%]	32 [3·5%, 2·5%−5·0%] #	0·736	2 [0·1%, 0·0%−0·3%]	4 [0·1%, 0·0%−0·2%]	0·689
Male	41 [2·7%, 2·0%−3·7%]	42 [1·7%, 1·2%−2·3%]	0·030	36 [2·5%, 1·8%−3·4%]	46 [1·9%, 1·4%−2·5%]	0·207	10 [0·2%, 0·1%−0·4%]	17 [0·1%, 0·1%−0·2%]	0·255
Age groups									
< 70 yrs.	21 [1·5%, 1·0%−2·3%] #	27 [1·1%, 0·8%−1·7%] #	0·361	13 [1·0%, 0·6%−1·7%] #	23 [1·0%, 0·7%−1·5%] #	0·937	6 [0·1%, 0·1%−0·2%]	10 [0·1%, 0·0%−0·1%] #	0·350
≥ 70 yrs.	40 [7·4%, 5·5%−10·1%]	51 [6·6%, 5·0%−8·6%]	0·555	40 [6·0%, 4·4%−8·1%]	55 [5·0%, 3·8%−6·5%]	0·398	6 [0·3%, 0·1%−0·7%]	11 [0·2%, 0·1%−0·4%]	0·457
CCI score									
0–2	7 [0·9%, 0·4%−1·8%] #	19 [1·2%, 0·8%−1·9%] #	0·457	2 [0·2%, 0·1%−0·9%] #	5 [0·3%, 0·1%−0·7%] #	0·753	3 [0·1%, 0·0%−0·3%]	3 [0·0%, 0·0%−0·1%] #	0·198
≥ 3	54 [4·8%, 3·7%−6·2%]	59 [3·8%, 3·0%−4·9%]	0·238	51 [4·5%, 3·4%−6·0%]	73 [4·3%, 3·4%−5·4%]	0·743	9 [0·2%, 0·1%−0·4%]	18 [0·2%, 0·1%−0·3%]	0·521

⁎ Data before January 24, 2019 in control period and before January 24, 2020 in study period was excluded.

# *P*<0·05, compared between different sexes, different age groups or different CCI score groups using Poisson models. **Abbreviations:** STEMI, ST-Elevation Myocardial Infarction; Non-STEMI, Non-ST-Elevation Myocardial Infarction; UAP, Unstable Angina Pectoris; CCI, Charlson Comorbidity Index.

**Figure. 3:**
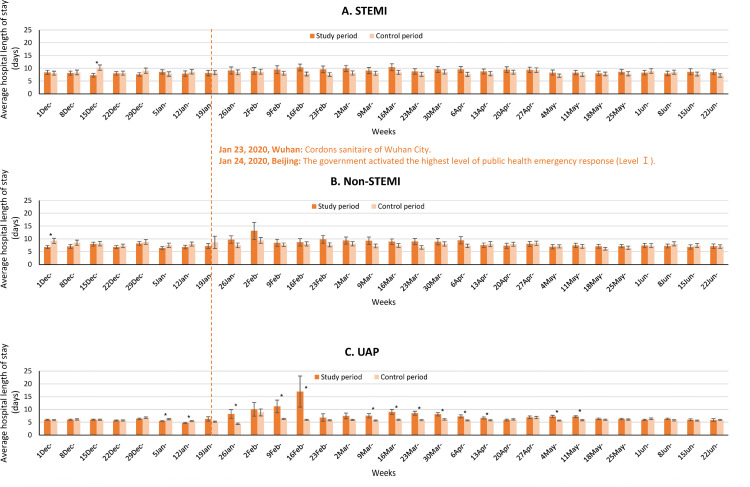
**Average Hospital Length of Stay (with Standard Error) for Admitted STEMI, Non-STEMI and UAP Patients by Weeks during Study Period or Control Period.** The weekly average hospital length of stay (LoS) was similar for admitted STEMI and Non-STEMI patients during study period and control period. However, the weekly LoS was higher during the first four months for admitted UAP patients, compared to the same period last year. *P<0·05, compared between study period and control period using *t-*test. **Abbreviations:** STEMI, ST-Elevation Myocardial Infarction; Non-STEMI, Non-ST-Elevation Myocardial Infarction; UAP, Unstable Angina Pectoris.

Average hospital length of stay (LoS) in patients with STEMI was 8·6 days in the study period and 8·2 days in the control period (*P* = 0·059). The corresponding numbers were 7·8 days and 7·6 days (*P* = 0·647) in patients with Non-STEMI, and 6·3 days and 5·9 days in patients with UAP (*P*<0·0001; Fig. 3).

**Figure. 4:**
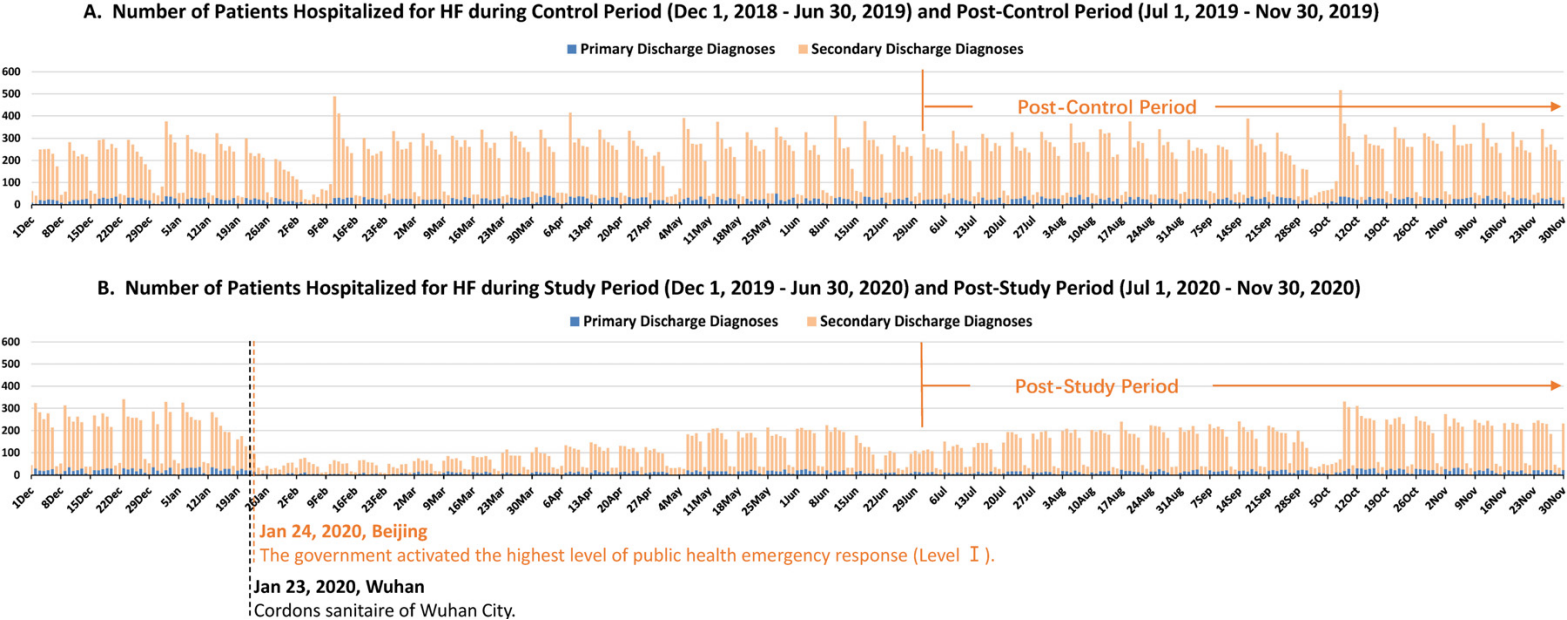
**Trends of Admissions for HF Patients during December 1, 2018 to November 30, 2019 and December 1, 2019 to November 30, 2020**. Compared to the control period, numbers of patients admitted with HF reduced remarkably since the government activated the highest level of public health emergency response (Level Ⅰ) on Jan 24, 2020. From July 1, 2020 to November 30, 2020, the admission numbers were still lower than numbers in equivalent time period in previous year (data between November 30, 2020 and January 24, 2021 were not shown). **Abbreviations:** HF, Heart Failure.

Number of patients admitted with HF was 13,775 in the study period, compared to 31,097 in the control period (55·7% lower; Fig. 4). During the 7 months following the study period (July 1, 2020 to January 23, 2021), there were 31,754 admissions for HF, compared to 41,736 admissions in equivalent time period in previous year (23.9% lower).

## Discussion

The current study showed significant declines in hospitalizations of STEMI, Non-STEMI, and UAP in Beijing from January 24, 2020, when the government announced the strictest response to public health emergency, to June 30, 2020, in comparison with the same time period in 2019. However, proportions of patients with ACS receiving PCI, proportion of patients with STEMI receiving PCI within 24 h, and proportion of patients with UAP receiving CABG were higher in the study period compared to those in the control period. In-hospital mortality rates for patients with STEMI, Non-STEMI, and UAP were not significantly different between the two periods. Subsequent HF admissions number was 23.9% lower in the 7 months post-study period compared to the equivalent time in the previous year.

Many countries took large-scale public health measures during the COVID-19 pandemic, such as stay-at-home orders, closing schools, cancelation of mass gathering, and even lockdown of endemic areas,^9^ which resulted in an unintentional negative effect on routine health management.^10^ Reduction in admitted patients presenting with ACS were observed globally after the outbreak of COVID-19.^1^, ^2^, ^3^,^5^,^11^,^12^ During the early phase since detection of the first COVID-19 positive case, STEMI admissions reduced by 23% in England,^2^ 25% in Western France,^11^ 24% in Northern Italy,^1^ and 25% in Austria,^3^ compared with the baseline that closed to outbreak point. In China, a reduction of 26% for STEMI admissions in the first month after lockdown of Wuhan in areas outside Hubei province (where Wuhan located in) was also reported, compared with the month before ^5^. In our study, from late January to June, during which time about 900 COVID-19 cases were detected in Beijing, number of patients hospitalized with STEMI decreased by 38%. Even after the Beijing government loosening the level of public health emergency response from Level Ⅰ to Level Ⅱ on April 30, 2020, the admissions for STEMI still stayed lower than that in the previous year, indicating the social effect of COVID-19 continued even though the pandemic had been quickly controlled. More dramatical drop of Non-STEMI admissions were reported in England, Northern Italy, Austria with a reduction of 42%, 44% and 49%, respectively.^1^,^2^,^11^ We observed a similar reduction of 41% for Non-STEMI admissions, and an even larger reduction of 63% for UAP admissions. These data suggest the reduction in ACS admission was driven by reduction in UAP admission.

Patients were reluctant to go to hospital unless their conditions were too severe during the COVID-19 pandemic. This was reflected by an older age and a higher CCI score for those admitted during the study period compared to those admitted during the control period. However, the in-hospital mortality was not significantly different between two periods, which partially attribute to a higher proportion of these high-risk patients received PCI procedures. This in time therapy may be due to the fact that elective PCI cases were reduced which reserve more space for physicians to do PCI for those who really need the treatment. This unexpected effect has an important implication as to how to modify the current health care system in the future. In China, PCI procedures increased 3-fold from 2006 to 2011 (age-standardized annual hospitalizations for PCI increased from 62,308 to 208,954),^13^ while hospitalization rates for any type of AMI just increased by 31.2% during similar period (from 55.8 per 100,000 in 2007 to 77.3 per 100,000 in 2012),^14^ which suggested the dramatically increased PCI procedures was driven by treating non-AMI. It might be time to reconsider whether this practice is optimal.

Our results are different from that reported by China Chest Pain Centers,^5^ where in-hospital mortality rates were higher in outbreak period than in control period among STEMI patients. There are several differences between these two studies that may explain the diversity: first, our study used an administrative database which included all the summary data of hospitalized patients; therefore, selection bias was fewer in our study. In contrast, the data of China Chest Pain Centers were reported by doctors which wasn't covered all admitted patients; Second, we only used data from hospitals located at Beijing, the capital city of China. The stable mortality rate may benefit from a more sufficient resource supply compared to other areas included in the China Chest Pain Centers.

It is still of important concerns as to whether the delay in seeking health care will lead to higher risk of cardiovascular mortality and HF hospitalization after the pandemic.^6^,^15^ Recent data from China's Disease Surveillance Points system outside Hubei province showed non-significant lower mortality rates of myocardial infarction (135·9 vs. 145·9 per 100,000) and hypertensive heart disease (24·4 vs. 25·6 per 100,000) in 2020 than routine predicted.^7^ In our study, we did not see any increase of HF admissions in Beijing after half year since the beginning of pandemic, instead, a fallen of 23.9% was observed in post-study period (July 1, 2020 to January 23, 2021) compared to the same time in last year (July 1, 2019 to January 23, 2020). The time lag of HF development after myocardial infarction is unclear, half year may be too short to observe the consequence of untimely treatment of STEMI patients. Also possible, the reduction in STEMI admission rate reflected a real decrease of incident STEMI during the study period due to less intensive workload and healthier lifestyle.

Twenty percent increase of death was estimated in other country overwhelmed by COVID-19, 33% of which attributed to causes other than COVID-19.^16^, ^17^, ^18^ China was not overwhelmed by burden of cardiovascular disease caused by the pandemic, which mostly benefited from the promote, active and strict strategies taken by the government and adequate personal protective equipment (PPE) provided to health care workers.^9^ However, there are still important issues to consider. Firstly, COVID-19 patients may develop ACS with much higher risk of mortality,^19^, ^20^, ^21^ and also might present with a ST-elevation on electrocardiogram, coronary spasm or myocardial injury without a documented Type Ⅰ or Type Ⅱ AMI,^22^, ^23^, ^24^ so that differential diagnosis is particularly important to make appropriate decisions on reperfusion therapy.^25^ Secondly, in case of admitting to non-PCI capable hospitals, fibrinolysis-based strategies could be considered in the first place,^26^ which was supported by the results of STREAM trial (Strategic Reperfusion Early After Myocardial Infarction) that early fibrinolysis could delay the time of PCI without reducing the effectiveness of reperfusion therapy.^27^ Thirdly, to maintain a balance between appropriate treatment for ACS patients and safety of health care workers, it is recommended that PPE should be in place for all medical staff taking care of ACS patients regardless of patients’ COVID-19 status.^25^

There are several limitations of our study. First of all, the nature of observational study design precludes causal inference, but trends of ACS admissions are congruent with other published studies. Second, because of incapable of acquiring out-hospital death information, we are not sure whether there was any significant change on total cardiac mortality during the pandemic in Beijing; however, HF admissions was used instead to indicate the short-term prognosis of ACS patients. Third, a significant increase of thrombolytics was previously reported in the first month since the outbreak,^5^ however, thrombolytic therapy is recorded in a separate medical system, so it is inaccessible in our study. Fourth, information of time to reperfusion therapy is temporarily unavailable in our database, but we used 24-hour PCI to represent the timeliness of PCI procedure, which was comparable with previous published studies.^2^,^5^ In addition, our findings based on local data couldn't represent for the whole country; however, the measures to interrupt transmission of COVID-19 varied significantly among different areas, it's inappropriate to pool data from different area together, and results from local data could be more interpretable.

In conclusion, although the COVID-19 pandemic was quickly under control, sharply reductions of ACS admissions were still happened in Beijing. But rates of cardiac procedures, including PCI and CABG, quickly got back to normal, and 24-hour PCI rate was even higher for STEMI patients in pandemic period. Furthermore, increase of HF admissions was not observed in post-study period, indicating the COVID-19 pandemic didn't deteriorate the short-term prognosis of ACS patients.

## Contributors

All authors contributed to study design, data interpretation, and writing of the report. LH, FL, XD, DL, CS, RT, JD, MG and CM had the idea for the study and contributed to study design. LH and XD analyzed data. LH, FL, XD, DL, CS, RT, JD, MG and CM interpreted data. LH, FL and XD wrote the draft report.

## Declaration of Competing Interest

Chang-Sheng Ma has received honoraria from Bristol-Myers Squibb (BMS), Pfizer, Johnson & Johnson, Boehringer-Ingelheim (BI) and Bayer for giving lectures. Jian-Zeng Dong has received honoraria from Johnson & Johnson for giving lectures. The remaining authors have no disclosures to report.

## Appendix Supplementary materials

Image, application 1

Image, application 2

## References

[1] De Filippo O., D'Ascenzo F., Angelini F. (2020). Reduced Rate of Hospital Admissions for ACS during Covid-19 Outbreak in Northern Italy. N Engl J Med.

[2] Mafham M.M., Spata E., Goldacre R. (2020). COVID-19 pandemic and admission rates for and management of acute coronary syndromes in England. Lancet.

[3] Metzler B., Siostrzonek P., Binder R.K., Bauer A., Reinstadler S.J. (2020). Decline of acute coronary syndrome admissions in Austria since the outbreak of COVID-19: the pandemic response causes cardiac collateral damage. Eur Heart J.

[4] Campanile A., Verdecchia P., Ravera A. (2021). Intensive cardiac care unit admission trends during the COVID-19 outbreak in Italy: a multi-center study. Intern Emerg Med.

[5] Xiang D., Xiang X., Zhang W. (2020). Management and Outcomes of Patients With STEMI During the COVID-19 Pandemic in China. J Am Coll Cardiol.

[6] Nicholls M. (2020). COVID-19 and cardiovascular disease. Eur Heart J.

[7] Liu J., Zhang L.Y., Zhou Y (2021). Excess mortality in Wuhan city and other parts of China during the three months of the covid-19 outbreak: findings from nationwide mortality registries. BMJ.

[8] Yurkovich M., Avina-Zubieta J.A., Thomas J., Gorenchtein M., Lacaille D. (2015). A systematic review identifies valid comorbidity indices derived from administrative health data. J Clin Epidemiol.

[9] Li Z., Chen Q., Feng L. (2020). Active case finding with case management: the key to tackling the COVID-19 pandemic. Lancet.

[10] Siegler J.E., Zha A.M., Czap A.L. (2021). Influence of the COVID-19 Pandemic on Treatment Times for Acute Ischemic Stroke: the Society of Vascular and Interventional Neurology Multicenter Collaboration. Stroke.

[11] Range G., Hakim R., Motreff P. (2020). Where have the ST-segment elevation myocardial infarctions gone during COVID-19 lockdown?. Eur Heart J Qual Care Clin Outcomes.

[12] Garcia S., Albaghdadi M.S., Meraj P.M. (2020). Reduction in ST-Segment Elevation Cardiac Catheterization Laboratory Activations in the United States During COVID-19 Pandemic. J Am Coll Cardiol.

[13] Zheng X., Curtis J.P., Hu S. (2016). Coronary Catheterization and Percutaneous Coronary Intervention in China: 10-Year Results From the China PEACE-Retrospective CathPCI Study. JAMA Intern Med.

[14] Zhang Q., Zhao D., Xie W. (2016). Recent Trends in Hospitalization for Acute Myocardial Infarction in Beijing: increasing Overall Burden and a Transition From ST-Segment Elevation to Non-ST-Segment Elevation Myocardial Infarction in a Population-Based Study. Medicine (Baltimore).

[15] Bahit M.C., Kochar A., Granger C.B. (2018). Post-Myocardial Infarction Heart Failure. JACC Heart Fail.

[16] Kittleson M.M. (2020). The Invisible Hand - Medical Care during the Pandemic. N Engl J Med.

[17] Katsoulis M., Gomes M., Lai A.G. (2021). Estimating the Effect of Reduced Attendance at Emergency Departments for Suspected Cardiac Conditions on Cardiac Mortality During the COVID-19 Pandemic. Circ Cardiovasc Qual Outcomes.

[18] Woolf S.H., Chapman D.A., Sabo R.T., Weinberger D.M., Hill L., Taylor D.D.H. (2020). Excess Deaths From COVID-19 and Other Causes, March-July 2020. JAMA.

[19] Madjid M., Safavi-Naeini P., Solomon S.D., Vardeny O. (2020). Potential Effects of Coronaviruses on the Cardiovascular System: a Review. JAMA Cardiol.

[20] Shi S., Qin M., Shen B. (2020). Association of Cardiac Injury With Mortality in Hospitalized Patients With COVID-19 in Wuhan, China. JAMA Cardiol.

[21] Bonow R.O., Fonarow G.C., O'Gara P.T., Yancy C.W (2020). Association of Coronavirus Disease 2019 (COVID-19) With Myocardial Injury and Mortality. JAMA Cardiol.

[22] Tam C.F., Cheung K.S., Lam S. (2021). Impact of coronavirus disease 2019 (COVID-19) outbreak on outcome of myocardial infarction in Hong Kong. Catheter Cardiovasc Interv.

[23] Guo T., Fan Y., Chen M. (2020). Cardiovascular Implications of Fatal Outcomes of Patients With Coronavirus Disease 2019 (COVID-19). JAMA Cardiol.

[24] Inciardi R.M., Lupi L., Zaccone G. (2020). Cardiac Involvement in a Patient With Coronavirus Disease 2019 (COVID-19). JAMA Cardiol.

[25] Mahmud E., Dauerman H.L., Welt F.G.P. (2020). Management of Acute Myocardial Infarction During the COVID-19 Pandemic: a Position Statement From the Society for Cardiovascular Angiography and Interventions (SCAI), the American College of Cardiology (ACC), and the American College of Emergency Physicians (ACEP). J Am Coll Cardiol.

[26] Bainey K.R., Bates E.R., Armstrong P.W. (2020). ST-Segment-Elevation Myocardial Infarction Care and COVID-19: the Value Proposition of Fibrinolytic Therapy and the Pharmacoinvasive Strategy. Circ Cardiovasc Qual Outcomes.

[27] Armstrong P.W., Gershlick A.H., Goldstein P. (2013). Fibrinolysis or primary PCI in ST-segment elevation myocardial infarction. N Engl J Med.

