# Supplementary material for: Impact of COVID-19 Pandemic on Hospital Admissions of Acute Coronary Syndrome: A Beijing Inpatient Database Study
Source: Lancet Reg Health West Pac. 2021 Dec 11;19:100335. doi: 10.1016/j.lanwpc.2021.100335 (PMC8665660; doi:10.1016/j.lanwpc.2021.100335)
Supplement: Supplementary file 1 [file mmc1.docx]

# Appendix

**Table 1 Numbers [Rates%] of Cardiac Procedures for Admitted STEMI, Non-STEMI and UAP Patients during Study Period and Control Period ^*^.**

|  | **STEMI** | | | **Non-STEMI** | | | **UAP** | | |
| --- | --- | --- | --- | --- | --- | --- | --- | --- | --- |
|  | **Study period** | **Control period** | ***P***  **value** | **Study period** | **Control period** | ***P***  **value** | **Study period** | **Control period** | ***P***  **value** |
| **PCI** |  |  |  |  |  |  |  |  |  |
| All | 1107/1953 [56·7%] | 1626/3150 [51·6%] | <0·0001 | 890/1991 [44·7%] | 1389/3373 [41·2%] | 0·012 | 3187/7664 [41·6%] | 8255/20868 [39·6%] | 0·002 |
| Sex |  |  |  |  |  |  |  |  |  |
| Female | 238/429 [55·5%] | 316/638 [49·5%] | 0·057 | 198/532 [37·2%] | 330/905 [36·5%] | 0·775 | 849/2330 [36·4%] | 2285/6591 [34·7%] | 0·124 |
| Male | 869/1524 [57·0%] | 1310/2512 [52·1%] | 0·003 | 692/1459 [47·4%] | 1059/2468 [42·9%] | 0·006 | 2338/5334 [43·8%] | 5970/14277 [41·8%] | 0·014 |
| Age groups |  |  |  |  |  |  |  |  |  |
| < 70 yrs. | 839/1415 [59·3%] | 1247/2373 [52·5%] | <0·0001 | 684/1321 [51·8%] | 1026/2274 [45·1%] | <0·0001 | 2438/5601 [43·5%] | 6370/15307 [41·6%] | 0·013 |
| ≥ 70 yrs. | 268/538 [49·8%] | 379/777 [48·8%] | 0·712 | 206/670 [30·7%] | 363/1099 [33·0%] | 0·318 | 749/2063 [36·3%] | 1885/5561 [33·9%] | 0·049 |
| CCI score |  |  |  |  |  |  |  |  |  |
| 0-2 | 415/823 [50·4%] | 722/1608 [44·9%] | 0·010 | 403/865 [46·6%] | 723/1662 [43·5%] | 0·138 | 1508/3579 [42·1%] | 4293/10250 [41·9%] | 0·793 |
| ≥ 3 | 692/1130 [61·2%] | 904/1542 [58·6%] | 0·173 | 487/1126 [43·3%] | 666/1711 [38·9%] | 0·022 | 1679/4085 [41·1%] | 3962/10618 [37·3%] | <0·0001 |
| **24-hour PCI** |  |  |  |  |  |  |  |  |  |
| All | 741/1953 [37·9%] | 998/3150 [31·7%] | <0·0001 | 158/1991 [7·9%] | 321/3373 [9·5%] | 0·049 | 129/7664 [1·7%] | 731/20868 [3·5%] | <0·0001 |
| Sex |  |  |  |  |  |  |  |  |  |
| Female | 154/429 [35·9%] | 200/638 [31·3%] | 0·122 | 36/532 [6·8%] | 73/905 [8·1%] | 0·369 | 34/2330 [1·5%] | 183/6591 [2·8%] | <0·0001 |
| Male | 587/1524 [38·5%] | 798/2512 [31·8%] | <0·0001 | 122/1459 [8·4%] | 248/2468 [10·0%] | 0·080 | 95/5334 [1·8%] | 548/14277 [3·8%] | <0·0001 |
| Age groups |  |  |  |  |  |  |  |  |  |
| < 70 yrs. | 564/1415 [39·8%] | 758/2373 [31·9%] | <0·0001 | 129/1321 [9·8%] | 249/2274 [10·9%] | 0·264 | 106/5601 [1·9%] | 601/15307 [3·9%] | <0·0001 |
| ≥ 70 yrs. | 177/538 [32·9%] | 240/777 [30·9%] | 0·441 | 29/670 [4·3%] | 72/1099 [6·6%] | 0·051 | 23/2063 [1·1%] | 130/5561 [2·3%] | <0·0001 |
| CCI score |  |  |  |  |  |  |  |  |  |
| 0-2 | 269/823 [32·7%] | 400/1608 [24·9%] | <0·0001 | 61/865 [7·1%] | 166/1662 [10·0%] | 0·014 | 58/3579 [1·6%] | 456/10250 [4·4%] | <0·0001 |
| ≥ 3 | 472/1130 [41·8%] | 598/1542 [38·8%] | 0·119 | 97/1126 [8·6%] | 155/1711 [9·1%] | 0·684 | 71/4085 [1·7%] | 275/10618 [2·6%] | 0·002 |
| **CABG** |  |  |  |  |  |  |  |  |  |
| All | 32/1953 [1·6%] | 68/3150 [2·2%] | 0·192 | 79/1991 [4·0%] | 158/3373 [4·7%] | 0·217 | 1095/7664 [14·3%] | 2381/20868 [11·4%] | <0·0001 |
| Sex |  |  |  |  |  |  |  |  |  |
| Female | 7/429 [1·6%] | 10/638 [1·6%] | 0·934 | 14/532 [2·6%] | 46/905 [5·1%] | 0·025 | 260/2330 [11·2%] | 615/6591 [9·3%] | 0·011 |
| Male | 25/1524 [1·6%] | 58/2512 [2·3%] | 0·147 | 65/1459 [4·5%] | 112/2468 [4·5%] | 0·904 | 835/5334 [15·7%] | 1766/14277 [12·4%] | <0·0001 |
| Age groups |  |  |  |  |  |  |  |  |  |
| < 70 yrs. | 24/1415 [1·7%] | 48/2373 [2·0%] | 0·476 | 63/1321 [4·8%] | 112/2274 [4·9%] | 0·834 | 870/5601 [15·5%] | 1847/15307 [12·1%] | <0·0001 |
| ≥ 70 yrs. | 8/538 [1·5%] | 20/777 [2·6%] | 0·179 | 16/670 [2·4%] | 46/1099 [6·3%] | 0·046 | 225/2063 [10·9%] | 534/5561 [9·6%] | 0·091 |
| CCI score |  |  |  |  |  |  |  |  |  |
| 0-2 | 16/823 [1·9%] | 26/1608 [1·6%] | 0·558 | 40/865 [4·6%] | 72/1662 [4·3%] | 0·735 | 532/3579 [14·9%] | 1167/10250 [11·4%] | <0·001 |
| ≥ 3 | 16/1130 [1·4%] | 42/1542 [2·7%] | 0·022 | 39/1126 [3·5%] | 86/1711 [5·0%] | 0·047 | 563/4085 [13·8%] | 1214/10618 [11·4%] | <0·001 |

*Data before January 24, 2019 in control period and before January 24, 2020 in study period was excluded.

**Abbreviations:**

STEMI, ST-Elevation Myocardial Infarction; Non-STEMI, Non-ST-Elevation Myocardial Infarction; UAP, Unstable Angina Pectoris; PCI, Percutaneous Coronary Intervention; CABG, Coronary Artery Bypass Grafting; CCI, Charlson Comorbidity Index.
