# Supplementary material for: Impact of COVID-19 Pandemic on Hospital Admissions of Acute Coronary Syndrome: A Beijing Inpatient Database Study
Source: Lancet Reg Health West Pac. 2021 Dec 11;19:100335. doi: 10.1016/j.lanwpc.2021.100335 (PMC8665660; doi:10.1016/j.lanwpc.2021.100335)
Supplement: Supplementary file 2 [file mmc2.docx]

**Appendix Table 1** Numbers [Rates%] of Cardiac Procedures for Admitted STEMI, Non-STEMI and UAP Patients during Study Period and Control Period ^*^.
